# Associations between estrogen receptor-beta polymorphisms and endometriosis risk: a meta-analysis

**DOI:** 10.1186/s13000-014-0184-x

**Published:** 2014-09-26

**Authors:** Renyong Guo, Nengneng Zheng, Shiping Ding, Ying Zheng, Limin Feng

**Affiliations:** Department of Laboratory Medicine, First Affiliated Hospital, College of Medicine, Zhejiang University, No. 79 Qingchun Road, Hangzhou, Zhejiang 310003 China; Department of Gynecology and Obstetrics, Tongde Hospital of Zhejiang Province, Hangzhou, Zhejiang 310012 China; State Key Laboratory for Diagnosis and Treatment of Infectious Disease, First Affiliated Hospital, College of Medicine, Zhejiang University, Hangzhou, 310003 China

**Keywords:** Endometriosis, Estrogen receptor-beta, Polymorphism, Meta-analysis

## Abstract

**Background:**

Many epidemiological studies have suggested an association between estrogen receptor-beta (ER-β) polymorphisms with endometriosis risk. However, the results of these studies have been inconsistent. In the present study, we performed a meta-analysis to clarify the associations between the ER-β rs4986938 and rs1256049 polymorphisms and endometriosis risk.

**Methods:**

Eligible publications were retrieved from the PubMed, ISI Web of Science, and several Chinese language databases. Pooled odds ratios (ORs) with 95% confidence intervals (CIs) were calculated using a random or fixed effect model.

**Results:**

A total of eight studies (1100 cases/1485 controls) for the rs4986938 polymorphism and four studies (353 cases/450 controls) for the rs1256049 polymorphism were included in this meta-analysis. Regarding the rs4986938 polymorphism, no obvious associations were found for all genetic models when all studies were pooled into the meta-analysis. In the subgroup analyses by ethnicity, study sample size, endometriosis-associated infertility, and stage of endometriosis, a significantly increased risk was observed among mixed populations (dominant model, OR = 2.03, 95% CI = 1.56–2.64) and among cases with endometriosis-associated infertility (dominant model, OR = 1.83, 95% CI = 1.26–2.67). Regarding the rs1256049 polymorphism, no obvious associations were found for all genetic models in the overall population. Subgroup analyses by ethnicity and study sample size revealed that only one study of a mixed population with small sample size showed an increased risk of endometriosis. No publication bias was found in the present study.

**Conclusions:**

The results of this meta-analysis suggest that the ER-β rs4986938 and rs1256049 polymorphisms may not be associated with endometriosis risk, while the observed increased risk of endometriosis-associated infertility may be due to bias by the inclusion of small-scale studies.

**Virtual Slides:**

The virtual slide(s) for this article can be found here: http://www.diagnosticpathology.diagnomx.eu/vs/13000_2014_184

## Background

Endometriosis is a steroid-dependent condition defined by the presence of endometrial tissue outside of the uterine cavity resulting in diverse clinical manifestations, such as infertility, pelvic pain, and dysmenorrhea [1]. Endometriosis affects 3%–10% of reproductive-aged women and 20%–50% of women with infertility [2]. Susceptibility to endometriosis is dependent on a complex interaction of genetic, immunological, and hormonal factors [3-5]; however, the exact etiology and pathogenesis of the disease remain unclear.

Estrogen has been shown to play a critical role in the growth of endometriotic tissues [6]. By binding to the estrogen receptor (ER), estrogen triggers a broad array of tissue and organ-specific physiological responses. There are two isoforms of ERs, ER-α and ER-β, which exhibit distinct distribution patterns among differentiated cells and tissues [7]. With the binding of ligands, ERs are translocated to the nucleus to activate gene transcription. Previous studies have demonstrated that allelic variants of genes encoding for ER may be responsible for their action as modulators of the estrogenic response, and the polymorphisms of these genes have been postulated as candidate risk markers for a number of estrogen-dependent disorders, including endometriosis [8,9]. The ER-β gene is located on the long arm of chromosome 14 at locus 2 among subloci 23 and 24 (14q22–24). Two single nucleotide polymorphisms (SNPs) in the ER-β gene, rs4986938 and rs1256049, have been most frequently studied. The former SNP located in exon 8 gives rise to a G-A exchange at nucleotide 1730 and introduces a recognition site for *AluI*, whereas the latter results in a G-A exchange at nucleotide 1082 in exon 5 and creates a recognition site for *RsaI*. In recent years, many meta-analyses have reported associations of these two SNPs in the ER-β gene with several diseases, including prostate cancer [10], male infertility [11], Parkinson’s disease [12], osteoarthritis [13], and breast cancer [14].

To date, a great number of case–control studies have been conducted to investigate the associations between the ER-β rs4986938 and rs1256049 polymorphisms and endometriosis risk in humans. However, the results of published studies are inconclusive and even controversial [15-23], and the associations between ER-β gene polymorphisms and the risk of endometriosis-associated infertility remain unclear, which could be due to differences in the studied populations and insufficient statistical power. Thus, we conducted this meta-analysis to explore the exact associations between the rs4986938 and rs1256049 polymorphisms of the ER-β gene and risk of endometriosis.

## Methods

### Publication search

A systematic literature search of the PubMed, ISI Web of Science, and several Chinese language databases (Biomedical Literature Database, Chinese National Knowledge Infrastructure, and Chinese Wanfang Data) was carried out to identify studies involving the associations between the ER-β rs4986938 and rs1256049 polymorphisms and endometriosis risk. No language restrictions were applied. To search and include as many related studies as possible, we used different combinations of the following key words: (estrogen receptor beta or estrogen receptor β or ER-β) and (polymorphism or variant or mutation) and endometriosis. All references of the retrieved studies were also reviewed to identify other relevant publications. Review articles were also searched to find additional eligible studies. If more than one article was published using the same study data, only the study with largest sample size was included. The literature search was updated on June 20, 2014.

### Inclusion and exclusion criteria

Studies fulfilling the following selection criteria were included in this meta-analysis: (1) case–control design; (2) evaluation of the correlation of the two SNPs (rs4986938 and rs1256049) of the ER-β gene with endometriosis risk; (3) provision of sufficient data to calculate the odds ratio (OR) and its corresponding 95% confidence interval (CI). Studies were excluded if one of the following criteria existed: (1) no relevance to the rs4986938 and rs1256049 polymorphisms, ER-β, and endometriosis; (2) non-clinical; and (3) review articles or commentaries. When several studies with overlapping data were eligible, those with the smaller sample size or less reliability were excluded. Furthermore, studies without detailed information were excluded after efforts to extract data from the original paper or contact the corresponding authors failed.

### Data extraction

Two authors (Renyong Guo and Nengneng Zheng) independently assessed the quality of each study. Any disagreement was resolved by consensus. The following information was extracted: name of first author, year of publication, country of origin, ethnicity of the study population, number of cases and controls, mean age of subjects and controls, number of infertility cases, genotype distributions in cases and controls, and probability (*p*) value for the test of Hardy–Weinberg equilibrium (HWE) in controls. Discrepancies occurring during the process of study selection and data extraction were resolved by discussion with a third reviewer (Limin Feng) to arrive at a consensus on each item.

### Statistical analysis

The strength of an association between the two ER-β gene polymorphisms and endometriosis risk was measured by OR with its corresponding 95% CI. The pooled ORs were calculated for the following four genetic models: (1) allelic model: allele A vs. G, where A = variant allele and G = wild allele, (2) homozygote comparison: AA vs. GG, (3) heterozygote comparison: GA vs. GG, (4) dominant model: AA + GA vs. GG, and (5) recessive model: AA vs. GA + GG. The statistical significance of the pooled OR was assessed with the Z test and a *p* value < 0.05 was considered statistically significant. A chi-square-based Q test was conducted to measure the heterogeneity between eligible studies and the existence of heterogeneity was considered significant at *p* < 0.10 [24]. When between-study heterogeneity was absent, a fixed effect model (Mantel–Haenszel method) was used to pool the data from different studies [25]; otherwise, a random-effects model using the DerSimonian–Laird method was applied [26]. Subgroup analyses were also conducted based on ethnicity, study sample size, endometriosis-associated infertility, and stage of endometriosis. Sensitivity analyses were performed to identify the effect of each individual study on pooled results and to test the reliability of results by deleting a single study each time [27]. Begg’s test and Egger’s test were performed to determine the presence of publication bias with *p* < 0.05 considered statistically significant [28,29]. Data analysis was performed using STATA statistical software (version 12.0; Stata Corporation, College Station, TX, USA).

## Results

### Characteristics of eligible studies

**Figure 1:**
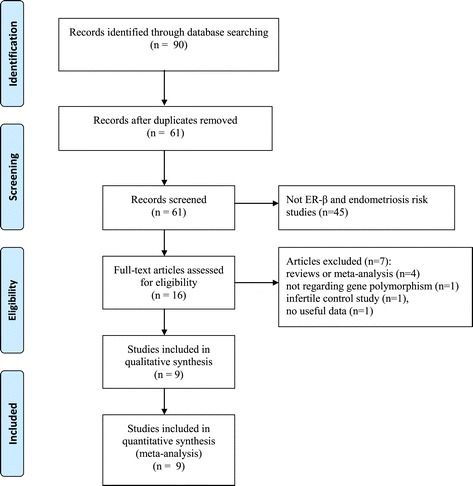
**Flow chart of publication selection.** A total of nine studies were included in this meta-analysis and systematic review after a comprehensive study selection.

The literature search identified a total of 90 potentially relevant papers. After removing 29 duplications and reading the titles and abstracts, a further 45 articles were excluded. A total of 16 remaining articles were then retrieved for further assessment of eligibility. Among these studies, seven were excluded because they were (1) reviews or meta-analysis [30-33], (2) not regarding gene polymorphisms [34], (3) an infertile control study [35], or (4) lacked useful data [36]. Finally, a total of nine eligible articles were included in this meta-analysis. The detailed screening process is shown in Figure 1, which was modified according to the Preferred Reporting Items for Systematic Reviews and Meta-Analysis (PRISMA) Statement [37]. Among these nine eligible studies, eight and four studies were pooled for analysis of the rs4986938 and rs1256049 polymorphisms, respectively. Regarding ethnicity, five studies assessed Asians [15-17,20,22] and four studies evaluated mixed ethnicities [18,19,21,23]. All of the included studies employed a case–control design and the genotype distributions among the controls of all studies were consistent with the HWE. The characteristics of the included studies are listed in Table 1.

**Table 1:** **Characteristics of the studies included in the meta-analysis of ER-β gene polymorphisms and endometriosis**

**Study**	**Year**	**Country**	**Ethnicity**	**Age, years, mean ± SD**		**Sample size**		**No. of infertility cases**	**Source of controls**	**Cases (Genotype)**			**Controls Genotype)**			***P***_**HWE**_^**b**^
				**Cases**	**Controls**	**Cases**	**Controls**			**G/G**	**G/A**	**A/A**	**G/G**	**G/A**	**A/A**	
*rs4986938*																
Wang [16]	2004	Japan	Asia	NA	NA	127	180	NA	HB	104	22	1	130	49	1	0.26
Huang [21]	2005	China	Asia	35.0 ± 10.0	34.0 ± 10.0	110	115	NA	PB	78	27	5	82	29	4	0.48
Lee [18]	2007	Korea	Asia	33.8 ± 6.7	41.6 ± 10.7	239	287	NA	HB	179	60	0	208	75	4	0.34
Bianco [19]	2009	Brazil	Mixed	34.1 ± 4.2	39.8 ± 4.5	108	210	108	HB	55	51	2	156	51	3	0.61
Zulli [20]	2010	Brazil	Mixed	33.7 ± 4.0	39.8 ± 4.5	136	209	136	HB	82	52	2	162	44	3	0.99
Gu [17]	2012	China	Asia	45.0 ± 6.0	40.0 ± 19.0	58	107	NA	HB	47	11	0	80	27	0	0.14
Christofolini [22]	2011	Brazil	Mixed	35.2 ± 3.9	34.4 ± 4.7	201	206	201	HB	123	76	2	145	58	3	0.30
Wu [23]	2013	China	Asia	30.9 ± 6.3	29.1 ± 4.3	121	171	121	NA	97	24^a^	NA	140	31^a^	NA	>0.05
*rs1256049*																
Wang [16]	2004	Japan	Asia	NA	NA	131	182	NA	HB	71	56	4	98	73	11	0.59
Huang [21]	2005	China	Asia	35.0 ± 10.0	34.0 ± 10.0	110	115	NA	PB	17	55	38	30	61	24	0.49
Silva [24]	2011	Brazil	Mixed	32.5^c^	37.4^c^	54	46	27	NA	22	32	0	43	3	0	0.82
Gu [17]	2012	China	Asia	45.0 ± 6.0	40.0 ± 19.0	58	107	NA	HB	27	19	12	50	45	12	0.70

HB, hospital-based; PB, population-based; NA, not available; No., number.

^a^GA + AA; ^b^ *p* value for Hardy–Weinberg equilibrium in controls; ^c^Mean age, years.

### Quantitative synthesis

#### ER-β rs4986938 polymorphism

**Figure 2:**
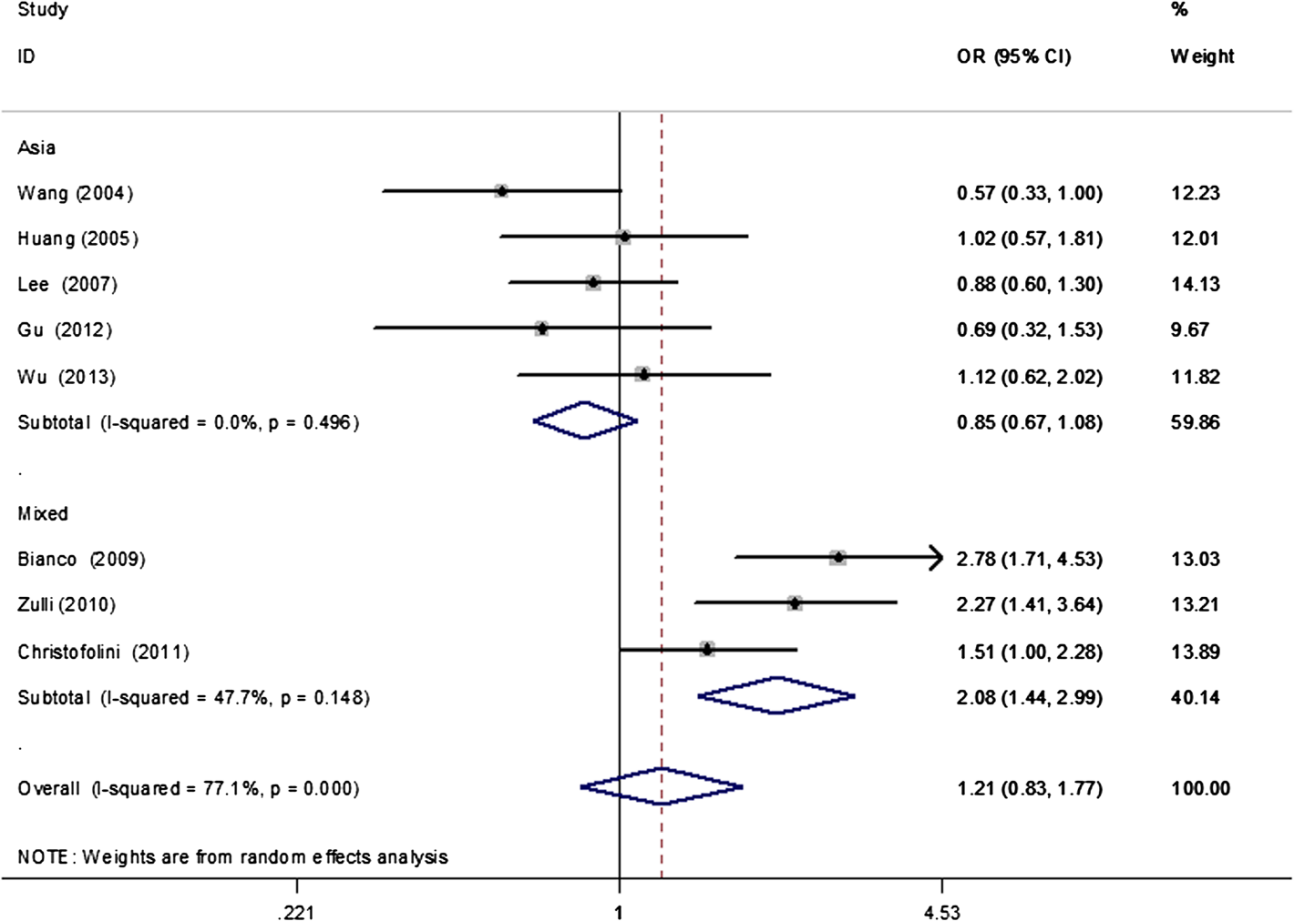
**Meta-analysis for the association of the ER-β rs4986938 polymorphism and endometriosis risk based on the dominant model (AA + GA vs. GG; stratified by ethnicity).**

**Figure 3:**
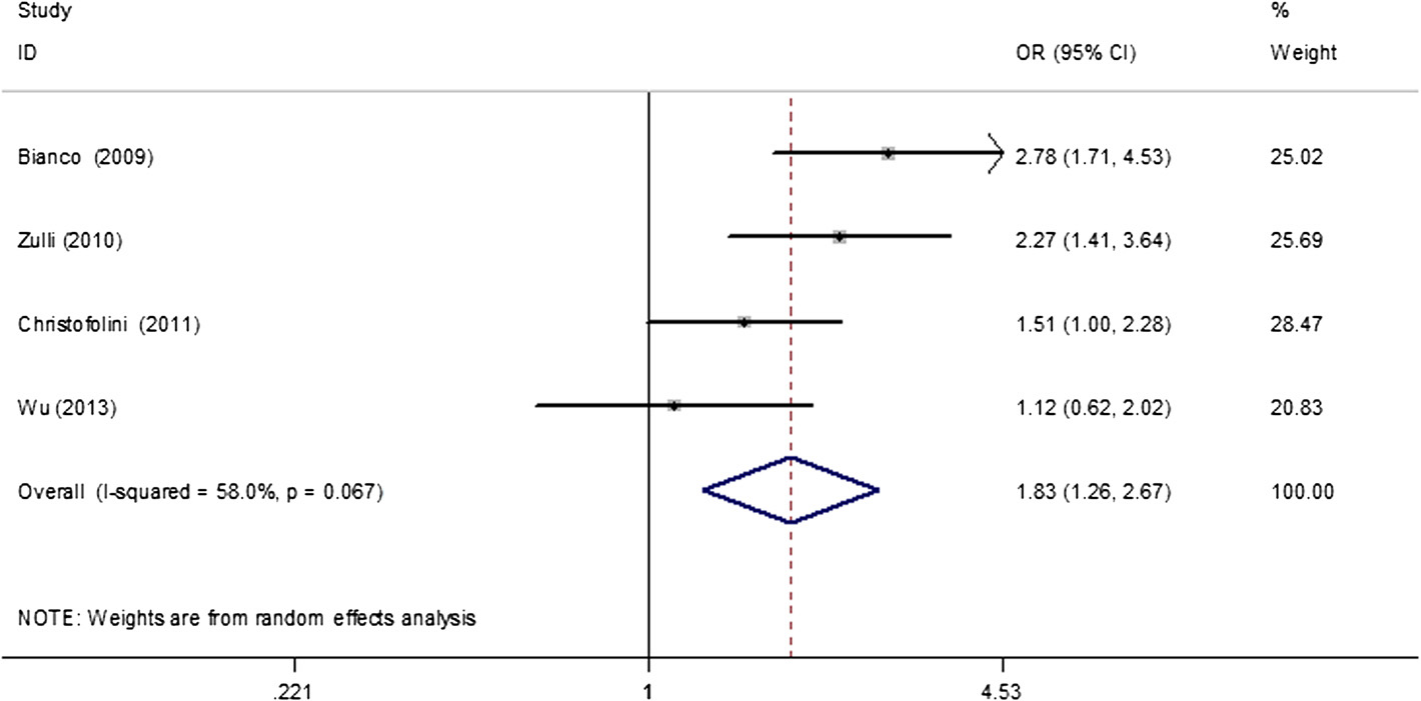
**A forest plot of the relationship of the ER-β rs4986938 polymorphism with the risk of endometriosis-associated infertility based on the dominant model (AA + GA vs. GG).**

**Figure 4:**
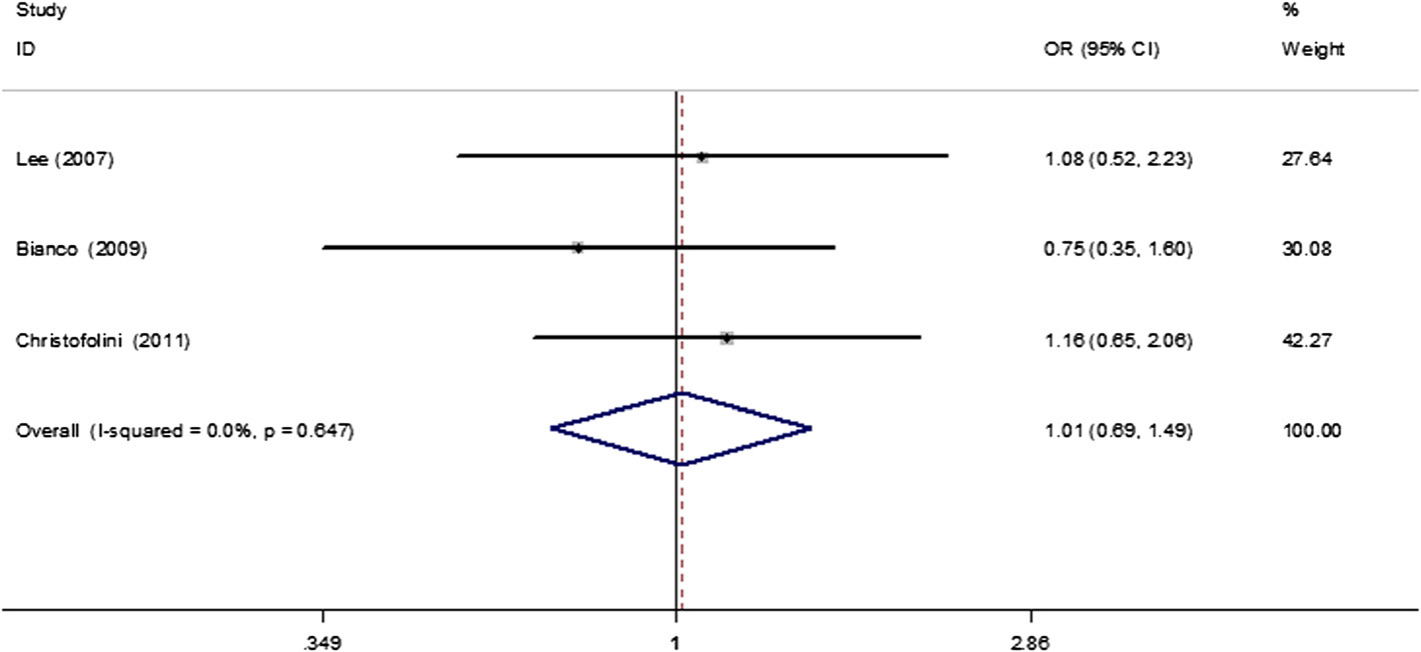
**A forest plot of the relationship of the ER-β rs4986938 polymorphism with stage of endometriosis (stage III–IV vs. stage I–II) based on the dominant model (AA + GA vs. GG).**

A total of seven studies with 1100 cases and 1485 controls were included to examine the association between the ER-β rs4986938 polymorphism and endometriosis risk. The overall results suggested no statistically significant association of this polymorphism with endometriosis susceptibility (dominant model, OR = 1.21, 95% CI = 0.83–1.77; recessive model, OR = 0.86, 95% CI = 0.41–1.77; AA vs. GG, OR = 0.95, 95% CI = 0.46–1.97; GA vs. GG, OR = 1.24, 95% CI = 0.80–1.91; A vs. G, OR = 1.16, 95% CI = 0.82–1.64; Table 2). In regard to subgroup analysis by study sample size (≤300 and > 300 subjects), the non-associations in the above-mentioned genetic models remained. However, further subgroup analyses based on ethnicities demonstrated that the ER-β rs4986938 polymorphism was significantly associated with an increased risk of endometriosis in mixed populations (dominant model, OR = 2.03, 95% CI = 1.56–2.64; GA vs. GG, OR = 2.08, 95% CI = 1.59–2.71; A vs. G, OR = 1.72, 95% CI = 1.37–2.17; Table 2, Figure 2). Additionally, four studies (566 cases and 796 controls) [18,19,21,22] provided data between the rs4986938 polymorphism and risk of endometriosis-associated infertility. The subsequent subgroup analysis showed that the ER-β rs4986938 polymorphism was also significantly associated with an increased risk of endometriosis-associated infertility (dominant model, OR = 1.83, 95% CI = 1.26–2.67; recessive model, OR = 0.96, 95% CI = 0.34–2.72; AA vs. GG, OR = 1.23, 95% CI = 0.43–3.49; GA vs. GG, OR = 2.08, 95% CI = 1.59–2.71; A vs. G, OR = 1.72, 95% CI = 1.37–2.17; Figure 3). Data on genotype distributions of the rs4986938 polymorphism in patients with stage I–II and III–IV endometriosis were also available in three studies [17,18,21]. The results showed no significant association of the rs4986938 polymorphism with stage of endometriosis (stage III–IV vs. stage I–II) (dominant model, OR = 1.01, 95% CI = 0.69–1.49; recessive model, OR = 0.34, 95% CI = 0.05–2.29; AA vs. GG, OR = 0.33, 95% CI = 0.05–2.29; GA vs. GG, OR = 1.05, 95% CI = 0.71–1.54; A vs. G, OR = 0.98, 95% CI = 0.70–1.36; Figure 4).

**Table 2:** **Summary ORs and 95% CIs of the association between ER-β rs4986938 polymorphism and endometriosis**

**Comparison**	**No. of studies**	**A vs. G** ^**b**^		**AA vs. GG** ^**a,b**^		**GA vs. GG** ^**b**^		**AA + GA vs. GG**		**AA vs. GA + GG** ^**a,b**^	
		**OR (95% CI)**	***P***_**H**_	**OR (95% CI)**	***P***_**H**_	**OR (95% CI)**	***P***_**H**_	**OR (95% CI)**	***P***_**H**_	**OR (95% CI)**	***P***_**H**_
Overall	8	1.16 (0.82-1.64)^R^	<0.001	0.95 (0.46-1.97)^F^	0.735	1.24 (0.80-1.91)^R^	<0.001	1.21 (0.83-1.77)^R^	<0.001	0.86 (0.41-1.77)^F^	0.790
*ethnicity*											
Asia	5	0.82 (0.65-1.04)^F^	0.552	0.75 (0.27-2.07)^F^	0.335	0.81 (0.62-1.05)^F^	0.456	0.85 (0.67-1.08)^F^	0.496	0.91 (0.26-3.13)^F^	0.331
Mixed	3	**1.72 (1.37-2.17)** ^c ,F^	0.204	1.23 (0.43-3.49)^F^	0.795	**2.08 (1.59-2.71)** ^c ,F^	0.159	**2.03 (1.56-2.64)** ^c ,F^	0.148	0.97 (0.34-2.74)^F^	0.880
*Sample size*											
>300	5	1.26 (0.82-1.93)^R^	<0.001	0.83 (0.34-1.99)^F^	0.611	1.41 (0.82-2.40)^R^	<0.001	1.38 (0.81-2.34)^R^	<0.001	0.71 (0.30-1.73)^F^	0.717
≦300	3	0.94 (0.62-1.41)^F^	0.412	1.31 (0.34-5.07)	-	0.86 (0.53-1.39)^F^	0.498	0.97 (0.67-1.39)^F^	0.624	1.32 (0.35-5.06)	-

OR, odds ratio; CI, confidence interval; No., number; *P*_H_, *p* value of Q-test for heterogeneity test; R, random-effect model; F, fixed-effect model.

^a^The study by Gu et al. was not included since they presented 0 frequency of AA genotype in cases and controls.

^b^The study by Wu et al. was not included since they just presented the data on GG and GA + AA genotypes in cases and controls.

^c^Statistically significant results (in bold).

#### ER-β rs1256049 polymorphism

**Figure 5:**
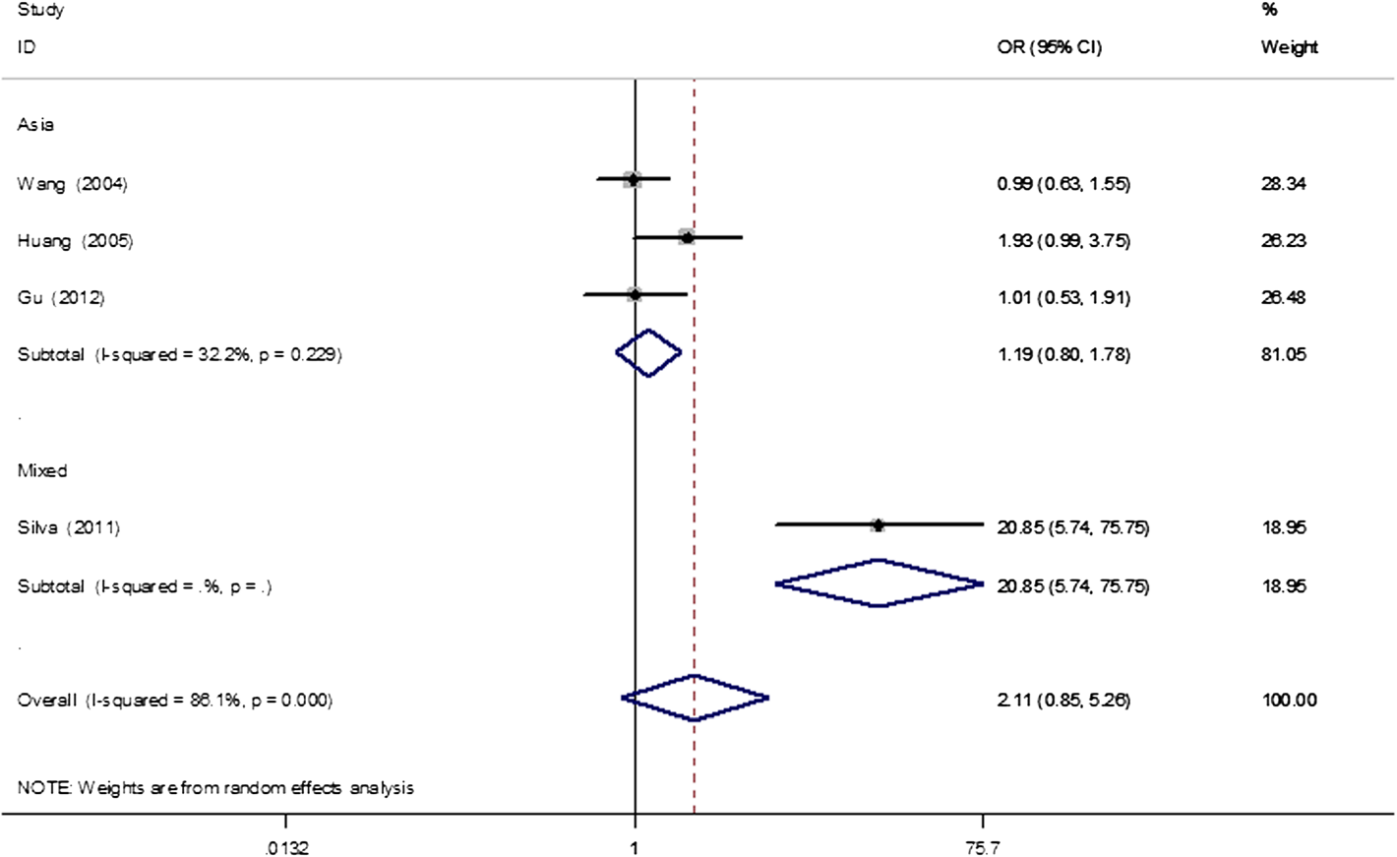
**Meta-analysis for the association of the ER-β rs1256049 polymorphism and endometriosis risk based on the dominant model (AA + GA vs. GG; stratified by ethnicity).**

A total of 353 cases and 450 controls were identified to assess the association between the rs1256049 polymorphism and endometriosis risk. The overall results suggested no statistically significant association of this polymorphism with endometriosis susceptibility (dominant model, OR = 2.11, 95% CI = 0.85–5.26; recessive model, OR = 1.43, 95% CI = 0.66–3.08; AA vs. GG, OR = 1.50, 95% CI = 0.59–3.79; GA vs. GG, OR = 1.95, 95% CI = 0.75–5.09; A vs. G, OR = 1.71, 95% CI = 0.92–3.16; Table 3). Further subgroup analysis suggested that the effect size was only significant among mixed populations (dominant model, OR = 20.85, 95% CI = 5.74–75.75; GA vs. GG, OR = 20.85, 95% CI = 5.74–75.75; A vs. G, OR = 12.49, 95% CI = 3.68–42.41), but not among Asian populations under all genetic models (Figure 5). Subgroup analysis by study sample size (≤200 and > 200 subjects) revealed no obvious associations.

**Table 3:** **Summary ORs and 95% CIs of the association between ER-β rs1256049 polymorphism and endometriosis**

**Comparison**	**No. of studies**	**A vs. G**		**AA vs. GG** ^**a**^		**GA vs. GG**		**AA + GA vs. GG**		**AA vs. GA + GG** ^**a**^	
		**OR (95% CI)**	***P***_**H**_	**OR (95% CI)**	***P***_**H**_	**OR (95% CI)**	***P***_**H**_	**OR (95% CI)**	***P***_**H**_	**OR (95% CI)**	***P***_**H**_
Overall	4	1.71 (0.92-3.16)^R^	<0.001	1.50 (0.59-3.79)^R^	0.059	1.95 (0.75-5.09)^R^	<0.001	2.11 (0.85-5.26)^R^	<0.001	1.43 (0.66-3.08)^R^	0.089
*Ethnicity*											
Asia	3	1.23 (0.86-1.75)^R^	0.094	1.50 (0.59-3.79)^R^	0.059	1.09 (0.78-1.53)^F^	0.371	1.17 (0.85-1.61)^F^	0.229	1.43 (0.66-3.08)^R^	0.089
Mixed	1	**12.49 (3.68-42.41)** ^b^	-	-	-	**20.85 (5.74-75.75)** ^b^	-	**20.85 (5.74-75.75)** ^b^	-	-	-
*Sample size*											
>200	2	1.22 (0.69-2.16)^R^	0.03	1.26 (0.23-6.76)^R^	0.018	1.20 (0.82-1.76)^F^	0.341	1.22 (0.85-1.77)^F^	0.101	1.08 (0.28-4.27)^R^	0.035
≦200	2	3.68 (0.36-37.65)^R^	<0.001	1.85 (0.73-4.68)	-	3.86 (0.15-102.43)^R^	<0.001	4.35 (0.21-89.43)^R^	<0.001	2.07 (0.86-4.95)	-

OR, odds ratio; CI, confidence interval; No., number; *P*_H_, *P* value of Q-test for heterogeneity test; R, random-effect model; F, fixed-effect model.

^a^The study by Silva et al. was not included since they presented 0 frequency of AA genotype in cases and controls.

^b^Statistically significant results (in bold).

#### Heterogeneity test and sensitivity analysis

**Figure 6:**
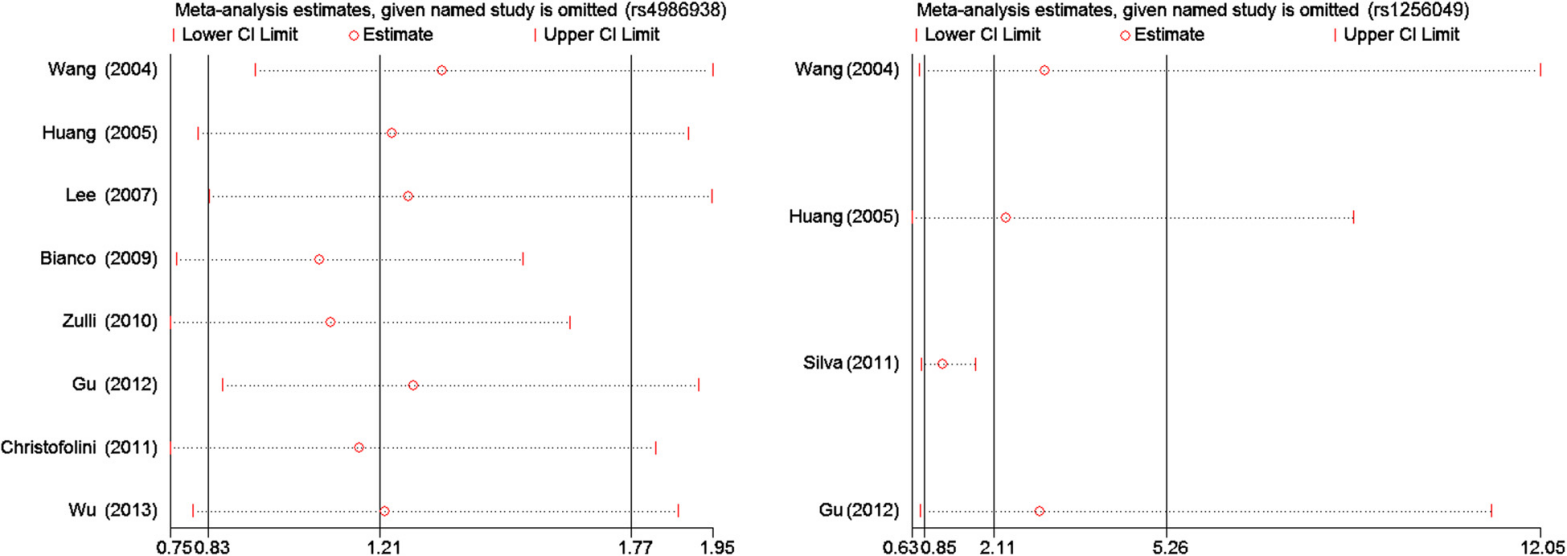
**Sensitivity analysis of the summary OR coefficients on the associations among the ER-β rs4986938 and rs1256049 polymorphisms with the risk of endometriosis based on the dominant model (AA + GA vs. GG).**

During the meta-analysis, significance of between-study heterogeneity was observed (Tables 2 and 3). To explore the source of heterogeneity, subgroup analyses by ethnicity and sample size were conducted. Furthermore, sensitivity analyses were performed to explore the influence of each individual study on the overall results by deleting a single study each time from the pooled analysis. This procedure confirmed that our results of the ER-β rs4986938 and rs1256049 polymorphisms and endometriosis susceptibility were both reliable and robust (Figure 6).

#### Potential publication bias

To assess possible publication bias in the currently available literature, Begg’s test and Egger’s test were performed, which showed no publication bias for studies published on the rs4986938 and rs1256049 polymorphisms (all *p* > 0.05, Table 4).

**Table 4:** **Statistical analyses of publication bias for ER-β gene polymorphisms**

**Category**	**A vs. G**	**AA vs. GG**	**GA vs. GG**	**AA + GA vs. GG**	**AA vs. GA + GG**
*rs4986938*					
Begg’s test	0.548	0.452	1.00	0.902	0.452
Egger’s test	0.452	0.211	0.507	0.493	0.205
*rs1256049*					
Begg’s test	0.308	0.296	0.734	0.089	0.296
Egger’s test	0.159	0.093	0.193	0.071	0.401

## Discussion

ERs are nuclear receptors that contain an estrogen-binding domain and a DNA-binding domain. Studies on tissue distribution and expression patterns indicate that ER-α has a broad expression pattern, whereas ER-β has a more focused pattern with high levels in the ovary, prostate, epididymis, lung, and hypothalamus [38]. Fazleabas et al. examined expression levels of ER-α, ER-β, and progesterone receptors in baboons and found that ER-β was the dominant steroid receptor present in endometriotic explants, regardless of the cycle phase [39]. Two meta-analyses demonstrated that the PvuII (T/C) and XbaI (A/G) polymorphisms of the ER-α gene may not be associated with endometriosis risk [30,40]. Over the last 10 years, a great number of studies have investigated the association between ER-β gene polymorphisms and endometriosis risk, with most focused on the ER-β rs4986938 and rs1256049 polymorphisms. However, some of the results were conflicting, even in the same population, and thus a systematic review and meta-analysis of associations between ER-β gene polymorphisms and endometriosis risk will be of great value.

To the best of our knowledge, this is the first meta-analysis to assess the relationship between the two ER-β gene polymorphisms and endometriosis risk across different ethnic populations and sample sizes. Even though the association of endometriosis risk and genetic polymorphisms involving biosynthesis of sex steroids and their receptors was assessed in a previous meta-analysis [30], the results only presented an allelic model of the ER-β rs4986938 and rs1256049 polymorphisms from five relevant studies. Our results suggested that there was no significant association between the rs4986938 and rs1256049 polymorphisms in the ER-β gene and endometriosis risk in the overall population, which was consistent with the results of a previous meta-analysis [30]. For the rs4986938 polymorphism, there was no statistically significant association among study sample size, stage of endometriosis, and Asian population in further subgroup analyses. However, obvious associations were found among mixed populations for rs4986938 polymorphisms under the allelic, heterogeneous co-dominant and dominant models. Different ethnic groups have unique genetic backgrounds and may produce variations of genetic factors involved in the pathogenesis of endometriosis [18]. The results of subgroup analyses indicated that ethnicity might be the source of heterogeneity across studies. However, a characteristic of three studies were mixed populations that included infertile Brazilian women with endometriosis. Therefore, the rs4986938 polymorphisms may be not associated with endometriosis risk, while the observed increase in the risk of endometriosis in mixed populations may be due to the complication of infertility. Regarding the rs1256049 polymorphism, no obvious associations were also found in all genetic models of the overall population and in subgroup analyses by study sample size. In the subgroup analyses based on ethnicity, however, only one study of a mixed population found an increased risk of endometriosis under allelic, heterogeneous co-dominant and dominant models of the rs1256049 polymorphism. Inherent genetic differences and variances in sample size between Asian and mixed population studies might be a potential explanation for the observed heterogeneity.

It should be noted that not all studies addressed the correlation of ER-β gene polymorphisms with the pathogenesis or risk of endometriosis-associated infertility. Regarding the rs4986938 polymorphism, only four studies evaluated the effect of interactions, with three studies [18,19,21] providing complete data on genotype distribution in cases and controls and one [22] just presenting data on GG and GA + AA genotypes in cases and controls. Regarding the rs1256049 polymorphism, since only one study [23] for endometriosis-associated infertility was investigated and the sample size was small, subgroup analysis of endometriosis-associated infertility was not performed. Nonetheless, the results demonstrated a significant association between the rs4986938 polymorphism and risk of endometriosis-associated infertility. Human fertility is a complex feature influenced by interactions between genetic and environmental factors. Saunders et al. reported that the ER-β gene plays an important role in the regulation of fertility in both males and females [41]. Another study performed with female ER-β knockout mice also demonstrated that ER-β proteins, which can be detected in multiple cell types throughout the female reproductive system, are essential for normal ovulation efficiency [42-44]. However, the functional significance of the rs4986938 polymorphism remains to be clarified. No amino acid changes in the ER-β protein occur under the conditions of ER-β gene rs4986938 mutations, whereas these polymorphisms are possibly in linkage disequilibrium with variations of other regulatory sequences that may affect gene expression or function [45]. Furthermore, structural folds of mRNA might change, accounting for the existence of SNPs, and could result in different biological functions that interact with other cellular components [46]. Endometriosis is noted in up to 20%–50% of infertile women, but the reason why women with endometriosis have impaired fertility remains uncertain [2]. The above reason might partly explain the potential association of polymorphic sites in the ER-β gene with endometriosis and endometriosis-associated infertility among different populations. However, it would be of great interest to characterize the actual relationship between ER-β gene polymorphisms and endometriosis and/or infertility in a large-scale study.

Several limitations to this study should be addressed. First, the results of our meta-analysis should be interpreted with caution because the sample size was relatively small for the subgroup analysis according to endometriosis-associated infertility and stage of endometriosis. Second, our meta-analyses were based on unadjusted estimates and therefore potential covariates, which might influence the effect estimates, were not controlled. Third, there were relatively few studies on the association of the rs1256049 polymorphism and endometriosis. Therefore, subgroup analysis according to endometriosis-associated infertility and stage of endometriosis was not performed. Hence, further studies with larger sample sizes to provide more detailed information are needed

## Conclusions

In conclusion, the results of this study suggest that the ER-β rs4986938 and rs1256049 polymorphisms may be not associated with endometriosis risk. However, ER-β rs4986938 is likely associated with endometriosis-associated infertility, and it might also act as a modifier of the relationship between risk of endometriosis-associated infertility and some environmental factors. To date, there is insufficient evidence implicating the ER-β rs4986938 polymorphism in the etiology of endometriosis-associated infertility for population testing. The observed increase in risk of endometriosis-associated infertility may be due to bias because of the inclusion of small-scale studies. It is critical that larger and well-designed multicenter studies should be performed to re-evaluate potential associations.
